# Role of α-Synuclein in the Prefrontal Cortex: From Physiological Synaptic Modulation to Synaptic Failure in Parkinson’s Disease

**DOI:** 10.3390/biomedicines14061394

**Published:** 2026-06-20

**Authors:** Uxia Argibay, María Sancho-Alonso, Claudia Yanes-Castilla, Judith Jericó-Escolar, Verónica Paz, Esther Ruiz-Bronchal, Lluis Miquel-Rio, Analia Bortolozzi

**Affiliations:** 1 Institute of Biomedical Research of Barcelona (IIBB), Spanish National Research Council (CSIC), 08036 Barcelona, Spain; uxia.argibay@iibb.csic.es (U.A.); maria.sancho-alonso@uv.es (M.S.-A.); claudia.yanes@iibb.csic.es (C.Y.-C.); judith.jerico@iibb.csic.es (J.J.-E.); veronica.paz@iibb.csic.es (V.P.); esther.ruiz@iibb.csic.es (E.R.-B.); lluis.miquel@iibb.csic.es (L.M.-R.); 2 Systems Neuropharmacology Research Group, Institut d’Investigacions Biomèdiques August Pi i Sunyer (IDIBAPS), 08036 Barcelona, Spain; 3 Biomedical Research Networking Center for Mental Health (CIBERSAM), Institute of Health Carlos III (ISCIII), 28029 Madrid, Spain; 4 Doctoral Program in Biomedicine, Universitat de Barcelona (UB), 08036 Barcelona, Spain; 5 Human Anatomy and Embryology Department, Faculty of Medicine, University of Valencia, 46010 Valencia, Spain; 6 Doctoral Program in Biochemistry, Molecular Biology and Biomedicine, Universitat Autònoma de Barcelona (UAB), 08193 Bellaterra, Spain

**Keywords:** α-Synuclein, dementia with Lewy bodies, glutamate, GABA, Parkinson’s disease, prefrontal cortex, synaptic plasticity

## Abstract

α-Synuclein (α-Syn) is a key presynaptic protein, primarily known for its role in the pathogenesis of Parkinson’s disease (PD) and other synucleinopathies, including dementia with Lewy bodies (DLB). Although much of the research has focused on the nigrostriatal dopamine (DA) pathway, there is growing recognition that the accumulation of misfolded α-Syn in the prefrontal cortex (PFC) is a critical driver of non-motor symptoms and cognitive deficits in PD and DLB. This review examines the dual role of α-Syn in the PFC circuitry, initially exploring its regulation of synaptic vesicle (SV) dynamics and recycling to maintain stable neurotransmission. We highlight its contribution to the modulation of glutamatergic (Glu) and GABAergic (γ-aminobutyric acid, GABA) synapses, which ensures the functional excitatory/inhibitory (E/I) balance of prefrontal circuits. Conversely, in PD and DLB, the transition of functional α-Syn monomers to pathological oligomers triggers a cascade of synaptic failures. We analyze how α-Syn aggregation causes pathology in dendritic spines, leads to a progressive reduction in the density of synaptic markers, and impairs cortical plasticity. Synthesizing evidence from neuroimaging studies, post-mortem human cortical samples, and animal models, this review emphasizes the PFC as a vulnerable brain region where α-Syn-mediated synaptic dysfunction translates into cognitive and emotional deficits. Deciphering these early synaptic alterations is essential for developing neuroprotective strategies that preserve cortical function in PD and DLB.

## 1. Introduction

α-Synuclein (α-Syn) protein, localized predominantly at the pre-synaptic terminals, is linked genetically and neuropathologically to Parkinson’s disease (PD) [1,2,3], as well as to a broader spectrum of α-synucleinopathies. Among these, Lewy Body Dementias (LBDs), encompassing dementia with Lewy bodies (DLB) and Parkinson’s disease dementia (PDD), primarily affect limbic and cortical networks, as well as brainstem regions [4,5,6]. Other clinicopathological entities exhibiting α-Syn pathology include incidental Lewy body disease (iLBD), which mainly affects neurons in the brainstem and limbic regions, and multiple system atrophy (MSA), characterized by oligodendrocyte and neuron involvement in the nigrostriatal and olivopontocerebellar domains. Additionally, Alzheimer’s disease (AD) with Lewy bodies presents as a complex mixed proteinopathy involving amyloid-β (Aβ), tau, α-Syn, and TDP-43, manifesting predominantly in limbic regions [6]. Although α-Syn pathology has widespread effects across these diverse phenotypes, this review focuses on the physiological and pathological roles of α-Syn within the prefrontal cortex (PFC). This anatomical region is particularly relevant to DLB, which accounts for approximately 30% of all age-related dementias [7], and to PD, where 20–40% of patients exhibit cognitive impairment at disease onset [8]. Cumulative longitudinal data suggest that up to 80% of PD patients will eventually develop PDD over the course of the disease [4,9], leading to a severe decline in the quality of life. Given that both conditions involve profound cognitive deficits affecting episodic, working memory, and executive functions [10,11,12], alongside motor and non-motor neuropsychiatric symptoms [12], disease-modifying therapies targeting cortical networks are critically desirable. Currently, no treatments can halt or reverse disease progression and cognitive deficits in DLB and PD, highlighting the urgent need for novel approaches targeting underlying pathophysiological mechanisms. While specific neuronal subtypes exhibit selective vulnerability in DLB and PD/PDD [13], substantial evidence suggests that synaptic dysfunction precedes overt neuronal loss and acts as a primary driver of cognitive decline [7,14,15]. Therefore, therapeutic interventions aimed at preserving and restoring synaptic integrity within these cortical circuits offer a promising strategy for arresting disease progression [16,17].

Both DLB and PD/PDD are characterized by neuronal α-Syn-positive intracytoplasmic inclusions, known as Lewy bodies and Lewy neurites. These inclusions also incorporate a variety of proteins and organelle components, including ubiquitin, tubulin, neurofilaments, lipids, and mitochondria markers [18]. Misfolded α-Syn oligomers are thought to act as prion-like proteins, propagating α-synucleinopathy by aggregation, intercellular spread, and the recruitment of endogenous monomeric α-Syn into pathological aggregates [19,20]. Beyond classic nigrostriatal dopaminergic (DA) loss, deficits in other brainstem monoaminergic pathways, including noradrenaline (NA) and serotonin (5-HT) neurons, have also been reported associated with PD [2,21,22,23,24]. More recently, the cognitive and neuropsychiatric profiles of PD, PDD, and DLB have been increasingly linked to the early involvement of the PFC, particularly the dorsolateral prefrontal (dlPFC) and ventromedial prefrontal (vmPFC) regions [25,26,27,28,29,30]. The PFC plays a key role in regulating cognitive plasticity, mood, and emotions [31,32,33]. Composed of 75–80% glutamatergic (glutamate, Glu) pyramidal projection neurons and 20–25% local circuit GABAergic interneurons (γ-aminobutyric acid, GABA), its functions depend to a large extent on its connectivity with a wide variety of other cortical and subcortical brain structures [34,35]. Recent paradigms regarding the spatiotemporal distribution of pathogenic α-Syn oligomers, such as the ‘body-first vs. brain-first’ hypothesis, suggest that α-Syn pathology may reach these cortical hubs via distinct pathways, either through ascending brainstem trajectories or via early cortical-limbic seeding [36,37,38]. For instance, recent preclinical studies suggest that α-Syn oligomer propagation leads to synaptic abnormalities in the PFC by promoting microglial-mediated synapse phagocytosis [17,39,40]. Despite its importance, our understanding of the physiological role of α-Syn in the PFC—particularly regarding the precise excitatory/inhibitory (E/I) balance essential for cognitive and emotional functions—remains limited, as does its pathological contribution to PD and related α-synucleinopathies. This review aims to dissect the mechanisms by which α-Syn regulates PFC synaptic plasticity. First, we examine the homeostatic role of α-Syn as a modulator of synaptic vesicle (SV) trafficking and recycling. We focus on its contribution to maintaining Glu and GABAergic synaptic integrity and neurotransmitter release, specifically within PFC circuits. Next, we address the transition to a pathological state, analyzing how the loss of α-Syn physiological function results in altered prefrontal synaptic plasticity and cytoarchitecture. Furthermore, we summarize recent clinical neuroimaging and neural network data linking these synaptic alterations to cognitive symptoms in PD, PDD, and DLB. We propose that targeting the PFC α-Syn–synapse interface offers a promising avenue for early diagnosis and the development of disease-modifying therapies. This approach could help preserve cognitive function and improve patient quality of life.

## 2. Physiological Role of α-Syn in PFC Glutamatergic and GABAergic Synapses

While not the primary focus of this review, we briefly summarize the anato-functional organization of the PFC. Located in the most rostral part of the frontal lobe, the PFC is considered the association cortex of this region. Although its anatomical boundaries are not strictly defined, it is characterized across all examined mammalian brains by its connectivity with the mediodorsal nucleus of the thalamus. According to Brodmann’s classification, the human PFC encompasses areas 8–14 and 44–47, though other frameworks also include ventromedial areas 14 and 25, which are deeply integrated in emotional networks. In humans, the vmPFC is a large, structurally heterogeneous region comprising several sub-regions involved in social cognition and emotion regulation; in contrast, dlPFC is primarily associated with cognitive control [32,41]. It is important to note that, while the vmPFC often refer to the entire ventromedial frontal lobes, only the more rostral regions are strictly prefrontal. The caudal aspects belong to the cingulate cortex (Cg), and proposed cross-species homology relate to these cingulate regions (area 24, 25 and 32). Indeed, areas 24, 25 and 32 have been identified in monkeys and rodents [42,43], although areas 24 and 32 exhibit more subdivisions in primates than in rodents. In the rodent brain, these regions correspond to the anterior cingulate (AC1/Cg1 and AC2/Cg2), prelimbic (PrL), and infralimbic (IL) cortices [32,43]. Based on cytoarchitectural studies, the IL is considered homologous to primate area 25, while the PrL corresponds to primate area 32 (see [32] for further details). Notably, these anatomical parallels are reflected in functional data; findings regarding α-Syn and synaptic stability in mouse models consistently mirror observations from neuroimaging studies and human post-mortem PFC samples [44].

α-Syn is a 140-amino-acid (~14.5 kDa) intrinsically disordered protein that plays a pivotal role in maintaining synaptic homeostasis [45]. Its inherent structural flexibility allows for rapid conformational transitions—most notably into amphipathic α-helices—upon binding to negatively charged phospholipid membranes or regions of high membrane curvature on SVs [46,47]. By binding to the outer leaflet of SVs, α-Syn influences the physical properties of the lipid bilayer, facilitating the vesicle clustering, docking, SNARE-complex formation, fusion, and endocytosis recycling at the presynaptic active zone. This biophysical interaction is not merely structural, but is linked to the energetics of SV fusion and the efficiency of the neurotransmitter release machinery [48,49,50,51].

Although the physiological role of α-Syn in the SV cycle has been extensively studied, its interactome continues to expand [52]. Early studies reported interactions between α-Syn and VAMP2 (known as synaptobrevin 2), a core component of the SNARE complex, as well as with β-and γ-synucleins, synapsin-I, synapsin-III, several proteins involved in calcium homeostasis, and many others [46,50,51]. A recent SV-omic study using isolated SVs from the brain homogenate of wild-type (WT), α-Syn-knockout, and α-Syn-transgenic mice (over 10 months of age) identified novel α-Syn interactors, including proteins and lipids. Prominent findings include alterations in SV proteins, such as ATP8A1, rab27B, synaptotagmin-1/2, SV2B, CADPS2, and NSF, among others. For instance, ATP8A1 is a P-type ATPase that regulates the interaction between α-Syn and synaptic proteins like synapsins and synaptogyrins to facilitate the formation of highly curved SVs [53]. Elucidating the α-Syn–SV interface requires a comprehensive set of tools to bridge the gap between molecular structure and biological function. Although high-resolution imaging and SV-omic studies have extensively mapped the α-Syn interactome, current functional understanding remains predominantly focused on DA synapses. Consequently, there is a notable paucity of data regarding the α-Syn synaptic mechanisms in non-DA circuits, even though the protein is widely expressed across various brain regions [53,54,55].

Both the propensity of α-Syn to aggregate and the progression of α-synucleinopathy depend on its local expression levels, its conformational state, and various post-translational modifications. Data from WT rodent models reveal a heterogeneous expression pattern, in which α-Syn is abundantly expressed in monoaminergic cells (DA, NA, 5-HT), but remains notably less abundant in cholinergic brain regions [55,56]. Interestingly, elevated α-Syn protein levels were also detected co-localizing with 5-HT-positive cells in the gastrointestinal tract of WT mice [57]. Despite this, surprisingly few studies have characterized α-Syn expression patterns within cortical regions. Previously, we reported high levels of α-Syn mRNA in WT mouse cortical areas, including PFC (both PrL and IL subdivisions), Cg, and motor cortices, alongside robust expression in subcortical structures, such as the hippocampus and monoaminergic nuclei (e.g., substantia nigra compacta—SNc, ventral tegmental area—VTA, locus coeruleus—LC, and raphe nuclei—RN) [58,59,60]. Supporting these findings, recent studies confirmed that α-Syn preferentially co-localizes with the excitatory Glu presynaptic marker, vesicular glutamate transporter-1 (VGLUT1), in the mouse PFC. VGLUT1 is mainly associated with intracortical Glu connections and long-range cortical Glu projections to subcortical areas, including amygdala and monoaminergic nuclei [61]. Conversely, α-Syn expression in GABAergic inhibitory synapses exhibits significant regional variability. While α-Syn protein co-localizes with glutamic acid decarboxylase (GAD) marker in inhibitory synapses of the olfactory bulb, globus pallidus, and substantia nigra pars reticulata, it is slightly expressed in GAD-positive synapses within the PFC and hippocampus [54,62]. These findings suggest a region-specific mechanism regulating α-Syn expression and function across different neuronal subtypes.

Studies conducted using primary cultures of rat cortical neurons have shown that exogenous α-Syn monomers mediate the mobilization of various SV reservoirs and the organization of active zone components at Glu synapses by binding to cholesterol. This interaction increases the tonic release of Glu, whilst reducing depolarization-induced release [63]. Furthermore, Glu neurons also express the glutamate vesicular transporter-2 (VGLUT2) [64]. Although α-Syn is generally more abundant in VGLUT1-positive terminals than in VGLUT2-positive ones, it also co-localizes with VGLUT2-positive neurons in specific brain regions, such as the thalamus [61,65]. Given the extensive thalamic projections to the PFC, this co-localization may have significant implications for cortical regulatory mechanisms.

It is worth noting that a subset of dopaminergic neurons in the VTA co-express VGLUT2, simultaneously releasing the neurotransmitters DA and Glu in cortical and subcortical regions [66]. These hybrid neurons exhibit resilience to various agents known to induce PD-related neurodegeneration, such as rotenone, MPTP (1-methyl-4-phenyl-1,2,3,6-tetrahydropyridine), and 6-OHDA (6-hydroxydopamine) [67]. Conversely, the deletion of VGLUT2 sensitizes these neurons to both MPTP [68] and 6-OHDA [67]. Supporting these findings, a recent study showed that exposure to toxic pre-formed α-Syn fibrils (PFFs) upregulates VGLUT2 levels in a model of Glu-induced human cortical neurons [69], suggesting that the VGLUT2-mediated response extends to broader neuronal populations. We have recently reported that the local α-Syn accumulation, including its phosphorylated forms, increases VGLUT2 levels in the mouse PFC [70]. These data provide key insights into cortical circuits and may enhance our understanding of PD-related cognitive deficits.

As has been observed in various types of synapses, α-Syn plays a key role in maintaining SV recycling pool size in both Glu and GABAergic cortical neurons [71]. Previous studies showed that both the recycling and total SV pools are more variable in Glu synapses than in their GABAergic counterparts [72]. This inherent heterogeneity could allow for a wider dynamic range of synaptic strength in excitatory neurons, a degree of plasticity that is significantly less pronounced in GABAergic synapses. Consequently, whereas α-Syn-mediated modulation of excitatory transmission is increasingly well-defined, its physiological role at inhibitory synapses in the PFC remains largely unexplored. Addressing this disparity is essential for a comprehensive understanding of how α-Syn regulates the delicate E/I balance of cortical circuit activity.

## 3. Pathological Role of α-Syn in PFC Glutamatergic and GABAergic Synapses

The physiological-to-pathological transition of α-Syn involves a complex conformational cascade where loss of normal synaptic function occurs concurrently with the emergence of neurotoxic reactive species. Although insoluble Lewy bodies enriched with phospho-α-Syn represent the definitive postmortem pathological hallmark of PD, PDD, and DLB, current evidence identifies soluble α-Syn oligomers as the main agents of immediate synaptic toxicity [73,74]. During the early phases of aggregation, these oligomeric assemblies disrupt cellular homeostasis through multiple concurrent mechanisms. They alter cell membrane permeability and perturb various organelles and RNA regulatory compartments, ultimately compromising nuclear stress pathways, endoplasmic reticulum (ER)–Golgi trafficking, mitochondrial dynamics, ER–mitochondria Ca^2+^ signaling, and endolysosomal degradation [6,75,76,77]. Concurrently, larger fibrillar conformers act as highly efficient templates for trans-synaptic seeding and propagation across interconnected neuroanatomical networks, driving the spatiotemporal progression of α-Syn pathology [78]. Importantly, these multi-scale pathological mechanisms do not occur in isolation; disease pathogenesis is further exacerbated by a broader landscape of systemic contributors. These include neuroinflammation and immune system dysfunction [79,80,81], metabolic and bioenergetic abnormalities [82,83,84,85], as well as environmental and lifestyle factors [86,87]. However, the precise mechanisms by which these factors converge to trigger the pathogenic transition of α-Syn are beyond the scope of this review.

As previously indicated, a disease model of PD based on α-Syn origin site and connectome (SOC model) has been proposed [37]. This model posits that α-Syn pathology initiates either in the olfactory bulb or amygdala—subsequently reaching cortical regions first and leading to a brain-first subtype—or in the enteric nervous system, resulting in a body-first subtype [37,38]. Postmortem mapping of α-Syn deposition in clinical cohorts consistently supports the anatomical validity of this top-down staging [36]. Crucially, the heterogeneous expression pattern of endogenous α-Syn is not merely a physiological baseline, but a critical factor in these divergent clinical trajectories [15]. In WT mice, α-Syn is abundantly expressed in brainstem monoaminergic neurons, as well as in gastrointestinal 5-HT-positive cells. Within the PFC, monomeric α-Syn preferentially colocalizes with VGLUT1 in excitatory projection neurons, while its presence at GABAergic synapses varies regionally. This cell-type and brain-region-specific α-Syn distribution provides a localized substrate that accelerates template-directed seeding into pathological aggregates. Therefore, the endogenous α-Syn expression map would serve as the key reference for determining whether the pathology progresses via ascending brainstem pathways (body-first) or thought early cortical/limbic seeding (brain-first) [15].

To experimentally model the cortical-predominant topography of the brain-first subtypes, recent paradigms have utilized stereotaxic injections of either α-Syn PFFs [88,89] or adeno-associated viral (AAV) vectors overexpressing human α-Syn [70,90,91] directly into the PFC of WT rodents. These approaches demonstrate that localized prefrontal α-Syn accumulation triggers a stereotyped, progressive rostro-caudal spreading of moderate-to-severe pathological aggregates across a distributed network, including the amygdala, hippocampus, and monoaminergic nuclei (the latter being particularly prominent in AAV-driven models) [70,88]. Importantly, this top-down propagation of α-Syn pathology yields significant functional consequences. Both male and female mice exhibit mild spatial working memory deficits and anxiety-like behaviors following cortical PFF or AAV challenges [70,88]. These findings underscore the PFC as a critical pathophysiological hub driving the non-motor prodromal and clinical manifestations of α-synucleinopathies.

Furthermore, recent circuit-level studies reveal that localized α-Syn aggregation affects cortical networks selectively, rather than uniformly. In a mouse model of intra-PFC α-Syn PFF infusion, pathological aggregation specifically impaired cortico-amygdala neurotransmission, while sparing adjacent thalamic–amygdala pathways [61]. This selective vulnerability aligns with baseline neurochemical profiles, in which prefrontal Glu projection neurons express high endogenous levels of VGLUT1, whereas thalamic projections preferentially express VGLUT2 [65]. The abundance of endogenous monomeric α-Syn in VGLUT1-positive neurons facilitates rapid molecular recruitment into aggregates. Consequently, electrophysiological recordings in mice following PFC-intra PFF injection reveal a significant reduction in the amplitude of evoked cortico-amygdala excitatory postsynaptic currents (EPSCs) [61], reflecting a functional dampening of excitatory neurotransmission. This phenotype is accompanied by a marked depletion of soluble α-Syn in VGLUT1-positive axon terminals, suggesting that the sequestration of α-Syn monomers into aggregates drives the enhanced short-term depression and compromised Glu release observed at these synapses [61]. Clinical studies support these findings, demonstrating that brain Glu neurotransmission is significantly attenuated in PD patients [92], alongside a reported reduction in VGLUT1 levels reaching up to 50% in both the temporal cortex and dlPFC [93,94].

In addition to local cortical injections, studies conducted in mice injected with α-Syn PFFs into the striatum also result in extensive α-Syn pathology in cortico-amygdala circuits [95,96,97]. In these models, VGLUT1-positive intracortical excitatory synapses display heightened vulnerability and undergo premature structural elimination. In contrast, inhibitory synapses positive for the vesicular GABA transporter (VGAT) remain relatively resilient in the PFC [98]. Nevertheless, emerging evidence suggests a nuanced, and sometimes conflicting, vulnerability profiles among specialized cortical GABAergic interneuron subpopulations. While parvalbumin (PV)-positive fast-spiking interneurons appear marginally affected by human WT α-Syn overexpression in the PFC of female mice, a potential compensatory increase in the density of somatostatin (SST)-positive interneurons has been observed [70]. Furthermore, translational assessments regarding the reorganization of the human postsynaptic inhibitory machinery remain scarce and warrant cautious interpretation. Preliminary studies using postmortem dlPFC and hippocampal samples from late-stage PD patients (Braak stages 5–6) have suggested elevated levels of the scaffold protein gephyrin (inhibitory postsynaptic GABA marker, personal communication). This contrasts with other cortical regions, such as the visual cortex of PDD and DLB patients, where significant reductions in gephyrin and GABA_A_ receptor-associated protein (GABARAP) have been reported compared to normal aging [99,100]. Taken together, these findings from preclinical and clinical models point to a highly heterogeneous and region-specific remodeling of the cortical GABAergic architecture linked to α-synucleinopathies. Given the current limitations and the scarcity of data in both humans and experimental models, rigorous mechanistic studies are needed to confirm these observations and understand their exact contribution to the cortical network dysfunction.

A major structural consequence of cortical α-Syn pathology is the disruption of dendritic spine architecture. The localized accumulation of α-Syn oligomers and larger aggregates is considered a primary driver of the profound dendritic spine loss observed in postmortem DLB brains [101,102]. Dendritic spines—highly regulated, small protrusions from the dendritic shaft—are essential for neuronal communication. They are extraordinarily abundant on pyramidal Glu neurons in the PFC, harboring the vast majority of excitatory synapses in the brain [103,104,105]. While early research in α-synucleinopathy focused predominantly on dendritic spine impairment within the striatum, hippocampus, olfactory bulb, and SNc [106,107,108,109], recent studies have identified clear prefrontal structural phenotypes [70,91,110,111]. Transgenic mouse models indicate that cortical α-Syn accumulation disrupts spine plasticity and density in the somato-sensorial cortex, with a 30% loss stabilizing after an initial decline at 3 months of age [110], thereby altering pre- and postsynaptic function. Building upon this, our group recently demonstrated that the progressive AAV-mediated accumulation of human α-Syn and its phosphorylated form in the PFC causes a significant loss of dendritic spine density at 8 and 24 weeks post-injection. This reduction specifically targets mushroom and stubby spines within deep cortical layers (L5/6) of female mice [70]. Conversely, longitudinal two-photon imaging has revealed that AAV-induced overexpression of human WT α-Syn prompts an initial, transient increase in cortical dendritic spine density around 5 weeks post-injection [91,111]. This structural expansion is driven by the prolonged survival of newly formed, persistent spines rather than accelerated spinogenesis or baseline elimination rates. Taken together, these data point to a biphasic, time-dependent structural progression in cortical α-synucleinopathy. It is highly plausible that early α-Syn accumulation triggers a transient, compensatory structural plasticity—manifesting as increased spine survival—which ultimately fails, leading to the severe, maladaptive spine pruning and synaptic loss observed at later stages of the disease.

Beyond alterations in structural and synaptic density, α-Syn pathology disrupts the molecular composition of the active zone and postsynaptic density (PSD) [15,44,112,113]. While presynaptic Glu impairments are well-characterized, a critical knowledge gap persists regarding the direct impact of cortical synucleinopathy on prefrontal postsynaptic ionotropic Glu receptors, specifically the α-amino-3-hydroxy-5-methyl-4-isoxazolepropionic acid (AMPA) subunits (e.g., GluA1/GluA2) and N-methyl-D-aspartate (NMDA) subunits (e.g., GluN1, GluN2A/B) [114]. Importantly, current mechanistic insights regarding postsynaptic Glu receptor vulnerability are derived almost exclusively from striatal and hippocampal models; therefore, they must be considered indirect evidence, rather than established cortical mechanisms. In these sub-cortical models, extracellular monomeric α-Syn physically destabilizes the lipid raft distribution of GluN2B subunits and PSD-95, disrupting their interaction and attenuating functional NMDA receptor-mediated currents [115]. Similarly, α-Syn oligomers block NMDAR-dependent long-term potentiation (LTP) in striatal spiny projection neurons (SPNs) [116]. Interestingly, AMPA receptors exhibit a different vulnerability profile in these circuits. Exposure of corticostriatal slices to either monomeric or oligomeric α-Syn preserves AMPA receptor-mediated miniature excitatory postsynaptic currents (mEPSCs) and baseline rectification indices, despite co-occurring NMDAR deficits [116]. Given the status of the PFC as the primary regulator of cognitive and psychiatric non-motor symptoms in PD/PDD and DLB, it is crucial to recognize these subcortical extrapolations as a starting point. Clarifying whether these exact postsynaptic receptor dynamics occur within prefrontal networks remains a fundamental requirement for designing targeted therapeutic strategies to reverse cortical circuit dysfunction.

It is important to note that, in patients with PDD and DLB, α-Syn pathology often co-exists with Aβ and tau accumulation, resulting in a complex mixed proteinopathy that accelerates cognitive decline and exacerbates executive dysfunction [6]. Recent experimental evidence highlights that this coexistence involves an active pathophysiological cross-talk. For instance, it has been demonstrated that early cortical Aβ deposition facilitates in vivo seeding and the spatiotemporal propagation of α-Syn pathology [117]. Furthermore, soluble Aβ oligomers may act as catalysts, directly triggering α-Syn aggregation through heterogeneous primary nucleation mechanisms [118]. Consequently, the synergistic interaction between Aβ, tau, and α-Syn in the PFC likely amplifies synaptic toxicity, promoting the dendritic spine loss and excitatory/inhibitory (E/I) imbalance that drives the clinical transition towards dementia.

## 4. A Comprehensive Analysis of SV2A Synaptic Loss, Prefrontal Connectivity Dysfunction, and Altered Excitatory/Inhibitory Balance in PD and DLB

Clinically, PD, PDD, and DLB have traditionally been distinguished using an arbitrary temporal criterion known as the one-year rule. This guideline separates these entities based on whether cognitive decline manifests before or more than one year after the onset of motor symptoms. However, current clinical evidence profoundly challenges this dichotomy, suggesting instead that these conditions represent phenotypic variants within a continuous spectrum of synaptic neurodegeneration and functional network disconnection [119,120,121]. In fact, despite sharing the core neuropathological hallmark of α-Syn aggregation into Lewy bodies and neurites, important biological distinctions separate these entities along the α-synucleinopathy spectrum. In PD without dementia, α-Syn pathology predominantly affects brainstem structures and the nigrostriatal pathway, driving motor symptoms while leaving cortical synaptic density relatively spared in the early stages. The transition to PDD involves a progressive spread of these aggregates into neocortical areas, corresponding with late-onset cognitive decline [2]. Conversely, DLB is characterized by an early, inherent cortical vulnerability, manifesting clinically as early-onset cognitive fluctuations, visual hallucinations, and parkinsonism. Furthermore, a critical distinguishing factor is the burden of concomitant proteinopathies. While pure α-Syn pathology can drive dementia, patients with DLB—and to a somewhat lesser extent, those with PDD—frequently exhibit a significantly higher burden of concurrent Aβ and tau pathology compared to PD patients [4,6,122]. This clinicopathological complexity highlights the immense challenge of accurately stratifying patients, as clinical symptoms often overlap and traditional assessments do not always reflect the precise underlying neuropathological stage or the specific cortical regions undergoing disconnection.

To address these stratification challenges, the incorporation of advanced molecular and functional neuroimaging has become indispensable. Positron emission tomography (PET) using ligands for the synaptic vesicle glycoprotein 2A (SV2A), such as ^11^C-UCB-J, has enabled the first in vivo quantification of synaptic density in clinical populations [123,124]. These studies demonstrate that synaptopathy is not a late-stage consequence, but an early event and a potent predictor of cognitive decline. In early-to-moderate PD without dementia, synaptic loss is predominantly localized to brainstem structures like the SN, where volumetric reductions can reach up to 50%, while cortical synaptic density remains relatively spared [125,126]. Conversely, PDD and DLB are marked by severe, widespread reduction in SV2A density across entire neocortical areas, including the frontal, parietal, temporal, and occipital lobes [127,128]. Intriguingly, in early-stage DLB, SV2A density within limbic structures, such as the hippocampus, is relatively preserved [129]. This distinct anatomical preservation explains a clinical observation in which episodic memory is often initially spared in DLB patients, contrasting sharply with the profound executive and visuospatial deficits driven by early neocortical synaptic loss.

Beyond measuring individual synaptic density, it is crucial to understand how the loss of presynaptic terminals alters large-scale dynamic communication across the brain. Resting-state functional magnetic resonance imaging (rs-fMRI) maps the functional connectivity by measuring the synchronization of low-frequency blood-oxygen-level-dependent (BOLD) signals between anatomically interconnected different brain regions. In patients with PD-associated mild cognitive impairment (PD-MCI), rs-fMRI reveals alterations in functional networks, predominantly affecting the dlPFC. Significant functional connectivity impairments occur between the dlPFC and posterior parietal cortices, as well as with subcortical structures, including the anterior putamen and the thalamus [125,130]. This progressive disruption of fronto-striato-thalamic loops and the fronto-parietal control network serves as the primary pathophysiological substrate for the executive dysfunction, bradyphrenia, and severe attentional deficits observed in PD-MCI [131,132]. Concurrently, a subset of PD-MCI patients exhibit anomalous patterns of hyper-connectivity or compensatory functional activation within contralateral prefrontal regions or specific nodes of the Default Mode Network (DMN). This transient increase in neural synchronization represents an endogenous neuroplastic attempt to mobilize auxiliary functional reserve and preserve cognitive integrity in response to localized synaptic depletion [132,133]. However, this compensatory mechanism is ultimately limited. As cortical synaptopathy exacerbates, evidenced by severe SV2A density reductions across the neocortex, these hyper-connected networks fail, marking the irreversible clinical transition from stable PD-MCI to widespread PDD and DLB.

The structural and functional organization of cortical circuits relies on a precise homeostatic balance between excitatory Glu and inhibitory GABA pathways, which can be monitored via focal metabolic changes using proton magnetic resonance spectroscopy (^1^H-MRS) [134,135]. Prefrontal Glu concentrations have emerged as reliable metabolic biomarkers for cognitive reserve. Although cognitively intact PD patients maintain stable PFC metabolic profiles, the onset of cognitive decline correlates with a progressive reduction in Glu or glutamine signals. Specifically, a significant reduction in the PFC Glu/creatine ratio (a marker of energy and metabolic reserve) has been reported in PDD patients [136], suggesting that PFC metabolic dysfunction occurs early in the dementing process. Interestingly, DLB exhibits more widespread Glu deficits than Alzheimer’s disease. In DLB, Glu depletion is not restricted to frontal areas, but extends into the posterior cingulate cortex, hippocampus, temporal lobes, and the caudate nucleus [137,138], matching the extensive visuospatial and attentional deficits seen in these patients.

In addition, inhibitory GABAergic signaling is critical for modulating PFC excitatory tone and shaping the high-frequency oscillatory synchronization required for executive tasks [139]. While GABA concentrations within the mPFC of PD patients often appear comparable to healthy controls [140,141], clinical stratification based on psychiatric comorbidities reveals striking differences. For instance, depressed PD patients exhibit a paradoxical increase in mPFC GABA levels compared to non-depressed PD patients. This finding contrasts with patterns observed in depressive disorder, where cortical GABA levels are diminished, suggesting that PD-related depression involves distinct pathophysiological mechanisms, such as localized interneuron hyper-reactivity or altered GABA receptor function [142]. Similarly, somatic symptom disorder and associated psychotic features in PD are linked to elevated mPFC GABA concentrations and altered functional connectivity [143]. These data suggest that excessive, maladaptive inhibitory tone in key prefrontal regions may impair reality processing and sensory integration, thereby contributing to neuropsychiatric symptoms in PD.

Despite its clinical utility, the in vivo tracking of cortical metabolites shows significant technical challenges. The low physiological concentration of GABA and its spectral overlap with Glu and glutamine resonances require specialized editing sequences, such as MEGA-PRESS (Meshcher–Garwood Point-RESOLVED Spectroscopy), and high-field magnetic resonance (≥3T) [144]. Furthermore, as metabolite levels fluctuate in response to stimuli or cognitive tasks, functional MRS (fMRS) has emerged as a vital tool to map dynamic neurotransmitter changes [145,146]. Ultimately, the field must transition toward multimodal neuroimaging integration. Combining fMRS, rs-fMRI, and SV2A PET imaging within the same cohorts offers a comprehensive framework to understand how localized molecular synaptopathy drives large-scale circuit desynchronization. Identifying patient subgroups based on their metabolic profiles (metabotypes) could facilitate targeted pharmacological interventions, such as NMDA receptor modulators or GABAergic agents, designed to restore synaptic homeostasis during the critical early windows of cognitive decline.

## 5. Advances in Cortical Neuroprotection for α-Synucleinopathies: Targeting Glutamate/GABA and Synaptic Stabilization via Ketamine and Psilocybin

The clinical and molecular understanding of PD, PDD, and DLB has undergone a fundamental paradigm shift, moving beyond a focus on subcortical dopaminergic degeneration toward a comprehensive cortical network perspective. However, the inherent heterogeneity of the clinical phenotypes, with DLB characterized by cognitive fluctuations, visual hallucinations, and early-onset parkinsonism, whereas PD typically progresses to dementia following years of motor symptoms, suggests distinct patterns of cortical vulnerability that require specific neuroprotective strategies [4,122]. Currently, the unmet need for disease-modifying therapies targeting cortical preservation remains critical. Although acetylcholinesterase inhibitors, such as donepezil and rivastigmine, represent the gold standard for managing cognitive symptoms in these disorders, their therapeutic impact is strictly symptomatic and temporary; they fail to arrest or reverse underlying synaptic atrophy [147]. Consequently, recent research has pivoted toward stabilizing PFC circuits by addressing the Glu and GABAergic imbalances that underpin cognitive and psychiatric symptoms. Ultimately, the goal of neuroprotection has expanded beyond simple cell survival to encompass the active maintenance of synaptic connectivity, structural remodeling, and homeostatic plasticity.

In this context, ketamine, traditionally utilized as an anesthetic and more recently as a rapid-acting antidepressant, has emerged as a potent neuroprotective therapeutic agent due to its ability to induce rapid neuroplasticity events [148,149,150]. Acting as a non-competitive Glu NMDA receptor antagonist, it triggers a transient Glu flow in the PFC, a phenomenon explained by the disinhibition hypothesis. By preferentially blocking NMDA receptors on GABAergic interneurons, ketamine diminishes inhibitory control over cortical pyramidal neurons, facilitating Glu release. The AMPA receptor-mediated activation subsequently engages the mammalian target of rapamycin (mTOR) signaling cascade and promotes brain-derived neurotrophic factor (BDNF) release [148,149,151]. Notably, recent preclinical findings from our group in mouse models of α-synucleinopathy demonstrate that the administration of sub-anesthetic doses of ketamine significantly upregulates both pre- (SV2A) and post-synaptic (PSD-95) protein levels, alongside a marked activation of the BDNF-TrkB signaling pathway within the mPFC [152,153]. This rapid induction of synaptic plasticity, occurring within a 12–24 h period, appears essential to counteract the functional connectivity deficits induced by α-Syn pathology [152].

Furthermore, psilocybin, a serotonergic psychedelic compound acting primarily as a 5-HT_2A_ receptor agonist, has gained significant attention for its ability to induce long-lasting neural plasticity after a single administration. Psilocybin appears to facilitate a plasticity window during which neural architecture undergoes remodeling through dendritic outgrowth and new synapse formation in the PFC and hippocampus [154,155]. The binding of psilocin (active metabolite) to cortical 5-HT_2A_ receptors recruits intracellular G_q/11_ and β-arrestin-2-dependent pathways, downstream engaging the BDNF and mTOR pathways, paralleling the effects of ketamine but via a different mechanism [156]. Longitudinal in vivo studies using two-photon microscopy evaluations in cortical tissue have shown that a single dose of psilocybin increases both the density and size of dendritic spines, directly strengthening synaptic efficacy [157]. In the context of Lewy body disorders, stimulating this structural remodeling within fronto-cortical and hippocampal networks offers a potential strategy to restore compromised hippocampal-prefrontal connectivity, which is critical for long-term memory retrieval and emotional regulation. Beyond structural cytoarchitectural repair, psilocybin exerts potent anti-inflammatory effects mediated by peripheral and central 5-HT_2A_ receptor activation [158,159]. Given that chronic neuroinflammation and glial activation are key drivers of progression in PD, PDD, and DLB [79,160], psilocybin’s ability to attenuate pro-inflammatory cytokine expression (e.g., TNFα, IL-1β) may provide a therapeutic dual advantage: reinforcing existing neural networks while shielding neurons from the proteotoxic environment that promotes α-Syn aggregation.

Reflecting these preclinical findings, several targeted clinical trials have been initiated to systematically evaluate the safety, tolerability, and preliminary efficacy of ketamine and psilocybin within synucleinopathy cohorts (Table 1). A significant milestone was the UCSF pilot study (NCT04932434), which represented the first investigation of a psychedelic in patients with confirmed neurodegenerative disease. While the results demonstrated that psilocybin significantly alleviated depressive and anxious symptoms—often refractory to conventional PD treatments—and yielded unexpected improvements in motor function, it is crucial to interpret these findings with caution. Researchers hypothesized that these motor gains might stem from enhanced plasticity within cortical-basal ganglia circuits and a reduction in systemic inflammation; however, this remains a mechanistic hypothesis. Similarly, trials such as the one conducted by Maastricht University (Eudra-CT2021-000041-40) are primarily designed to ascertain whether neuroplastic benefits can be harnessed without acute hallucinogenic effects, prioritizing tolerable dosing regimens (e.g., repeated low-dose 5 mg psilocybin and oral 35 mg ketamine) for an elderly population.

It must be emphasized that while these compounds demonstrate robust neuroplastic effects in experimental systems, definitive evidence for disease modification in human α-synucleinopathies is currently lacking. The available clinical data are derived predominantly from small pilot investigations focused on psychiatric symptoms, rather than the arrest of neurodegeneration. Nevertheless, integrating ketamine and psilocybin into the therapeutic landscape of α-synucleinopathies represents a conceptual shift from traditional monoamine-replacing therapies (e.g., dopaminergic supplementation) toward the exploration of active synaptic stabilization. In PD, PDD, and DLB, where the core pathophysiology involves a progressive failure of the structural and functional synaptic plasticity in cortical networks [161], we hypothesize that the potential therapeutic actions of these alternative psychoactive agents could operate across two synergistic levels. (1) Structural stabilization level: By potentially driving spinogenesis and dendritogenesis, these compounds might counteract the progressive dendritic atrophy and loss of post-synaptic densities induced by intracellular α-Syn oligomers, as observed in preclinical models. (2) Homeostatic level: By modulating NMDA and 5-HT_2A_ receptors on specific cellular sub-populations, these compounds could help restore the altered E/I balance within prefrontal networks. While recalibrating the microcircuit dynamic could improve cortical processing and address the mechanisms underlying cognitive fluctuations and executive inflexibility, large-scale, longitudinal clinical trials are strictly required to determine whether these preclinical neuroplastic properties can translate into true disease-modifying therapies for patients with advanced α-synucleinopathies.

## 6. Limitations and Critical Evaluation of Current Literature

Although the integration of molecular, structural, and neuroimaging data provides a comprehensive view of PFC α-Syn pathology, the reviewed literature presents specific methodological strengths and limitations that should be critically evaluated. These can be divided into preclinical experimental models and clinical studies.

Preclinical rodent models, particularly those utilizing stereotactic intra-PFC injections of PFFs or AAV vectors, offer the distinct advantage of establishing direct causality. They enable the precise mapping of spatiotemporal α-Syn spread and the selective isolation of specific vulnerabilities within prefrontal circuits, such as the VGLUT1-positive cortico-amygdala pathway. Furthermore, these models allow the longitudinal tracking of dendritic spine dynamics using two-photon microscopy, an approach currently unfeasible in humans. However, these models have certain limitations. First, the anatomical and functional homology of the PFC between rodents and humans remains a subject of debate. While the rodent PrL and IL cortices share similarities with primate cingulate and ventromedial regions, rodents lack the granular layer of dlPFC [162], a region severely affected in DLB and PDD [163]. Second, experimental models are often based on the acute overexpression of human α-Syn (via AAVs) or on massive bolus injections of PFFs, which may not accurately reflect the slow progressive kinetics of α-Syn aggregation characteristic of human aging. Finally, many experimental cohorts utilize young adult mice, thereby omitting the incorporation of the critical variable of advanced age and the associated glial senescence that inherently drives clinical α-synucleinopathies.

In the clinical setting, the advent of advanced neuroimaging techniques has provided significant advantages. SV2A-PET (e.g., ^11^C-UCB-J) and rs-fMRI allow for the in vivo quantification of synaptic density and the mapping of functional disconnections within fronto-striato-thalamic networks. Simultaneously, fMRS yields real-time metabolic biomarkers of the E/I balance by tracking prefrontal Glu fluctuations. Despite these advances, current neuroimaging approaches have notable weaknesses. The spatial resolution of SV2A-PET is currently insufficient to distinguish between presynaptic excitatory versus inhibitory terminal loss, or to resolve layer-specific cortical atrophy. Similarly, in vivo monitoring of GABA using ^1^H-MRS is technically challenging due to its low physiological concentration and spectral overlap with Glu and glutamine, requiring specialized sequences (e.g., MEGA-PRESS) and high-field magnetic resonance (≥3T), limiting its widespread clinical application. Furthermore, while current imaging techniques can monitor downstream synaptic loss, they cannot reliably visualize the underlying protein aggregates in vivo. To address this fundamental limitation, significant efforts are currently focused on the development and validation of highly specific PET tracers for α-Syn [164]. The ability to image α-Syn aggregates in the living brain would represent a major breakthrough, enabling the precise, longitudinal tracking of the α-synucleinopathy trajectory and its direct correlation with synaptic failure across different disease stages. Finally, while postmortem human studies provide definitive molecular validation, they predominantly reflect end-stage disease. These tissue samples are often affected by postmortem interval artifacts and by age-related co-pathologies, making it difficult to isolate of exclusively α-Syn-caused synaptic deficits.

## 7. Conclusions

**Figure 1:**
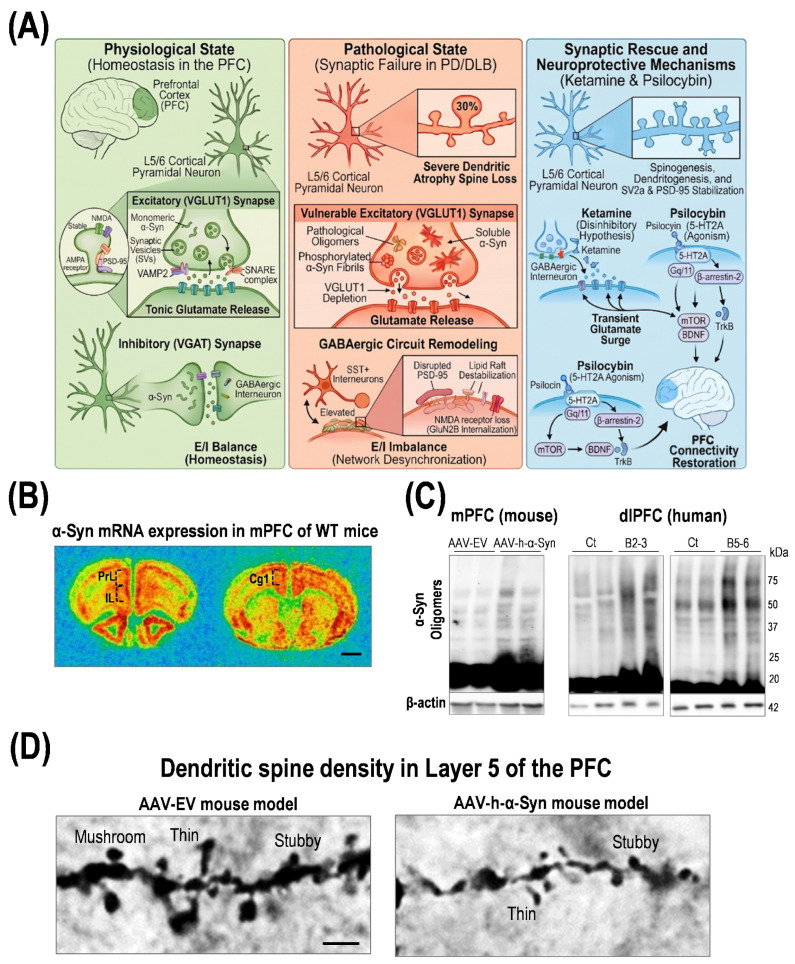
The dual role of α-Synuclein (α-Syn) in the prefrontal cortex (PFC) and therapeutic mechanisms for synaptic restoration. (**A**) Schematic overview of α-Syn protein function across physiological, pathological, and treatment states. Left (Physiological State): Monomeric α-Syn modulates synaptic vesicle dynamics to maintain stable neurotransmission at both excitatory (VGLUT1-positive) and inhibitory (VGAT-positive) synapses, preserving the functional excitatory/inhibitory (E/I) balance within prefrontal circuits. Center (Pathological State): The transition of α-Syn from a physiological to a pathological state is driven by the convergence of multiple cellular and systemic factors, including neuroinflammatory pathways, immunomodulation, metabolic stress, and impaired endolysosomal degradation, as well as the concurrence of complex mixed proteinopathies. In Parkinson’s disease (PD), Parkinson’s disease dementia (PDD), and Dementia with Lewy bodies (DLB), the accumulation of pathological α-Syn oligomers induces VGLUT1 depletion, severe dendritic spine loss, and the destabilization of PSD-95 protein and NMDA receptors. This cascade triggers GABAergic circuit remodeling, ultimately leading to E/I imbalance and network desynchronization. Right (Synaptic Rescue): Neuroprotective interventions with ketamine and psilocybin actively drive spinogenesis and dendritogenesis, counteracting the atrophy induced by α-Syn oligomers. Ketamine acts via the disinhibitory hypothesis by blocking NMDA receptors on GABAergic interneurons, triggering a transient glutamate release. Psilocybin acts as a 5-HT_2A_ receptor agonist. Both mechanisms converge on the downstream activation of mTOR and BDNF-TrkB signaling pathways, facilitating PFC connectivity restoration. (**B**) Coronal brain sections showing abundant α-Syn mRNA expression in the medial PFC (mPFC) of wild-type (WT) mice, specifically localized within the prelimbic (PrL), infralimbic (IL), and cingulate (Cg1) cortices assessed by in situ hybridization. Scale bar: 1 mm (adapted from [60]). (**C**) Western blot analysis of α-Syn protein in postmortem samples from human and mouse specimens. The left panel demonstrates increased oligomeric α-Syn in the mPFC of an adeno-associated viral (AAV-human-α-Syn)-induced overexpression mouse model compared to the empty vector (AAV-EV) control. The right panels show the presence of α-Syn oligomers in postmortem samples from dorsolateral PFC (dlPFC) tissue across progressive Braak stages (early B2-3 and late B5-6) compared to controls (Ct). β-actin serves as a loading control (adapted from [44]). (**D**) High-resolution imaging detailing dendritic spine density in Layer 5 (L5) of the PFC. AAV-h-α-Syn mouse model demonstrates a pronounced, maladaptive reduction in dendritic spines—particularly targeting the mushroom and stubby morphologies—when compared to AAV-EV controls. Scale bar: 5 μm (adapted from [70]). Created in BioRender. Bortolozzi, A. (2026) https://BioRender.com/f5solda, with the assistance of artificial intelligence.

The PFC is a critical hub where α-Syn acts as a vital physiological regulator of the E/I balance by modulating synaptic vesicle dynamics. This review highlights how the transition from functional monomers to toxic oligomers triggers early synaptic failures, including the selective loss of VGLUT1-positive Glu terminals and maladaptive spine pruning. Clinical imaging modalities (SV2A-PET, rs-fMRI, and fMRS) have confirmed that this cortical synaptopathy is a potent predictor of cognitive decline in PD, PDD, and DLB. Consequently, the therapeutic paradigm for these pathologies must shift away from late-stage monoaminergic neurotransmitter replacement toward early synaptic connectivity stabilization and neuroplastic preservation. Emerging therapies, such as ketamine and psilocybin, offer a promising neuroprotective frontier by promoting structural remodeling and recalibrating prefrontal network dynamics (Figure 1). Ultimately, unravelling the molecular interface that regulates α-synucleinopathy at the PFC synapse and validating multimodal biomarkers will be crucial to developing true disease-modifying strategies that preserve cognitive integrity and improve patient quality of life.

## Figures and Tables

**Table 1:** Current clinical trials involving ketamine and psilocybin in α-synucleinopathies.

Trial Identifier	Intervention	Target	Phase	Primary Objective	Status
NCT04944017	Ketamine (IV infusion)	PD and Depression	Phase 2	This study examines the efficacy and safety of a repeated-dosing ketamine infusion paradigm compared to placebo in individuals with PD. Utilizing advanced neuroimaging (PET and rs-fMRI), a subset of participants will be evaluated to determine whether ketamine’s antidepressant effects are driven by modifications in synaptic density and the functional reorganization of neural networks.	Completed
NCT06231563	Ketamine (Single dose)	Veterans with PD	Phase 2	This randomized, placebo-controlled study examines whether an intravenous (IV) dose of ketamine improves depression in Veterans with PD. Additionally, ketamine’s effects on underlying neuroplasticity and inflammatory pathways will be evaluated.	Recruiting
NCT04932434	Psilocybin (Oral)	PD and Depression/Anxiety	Pilot	The purpose of this study is to determine the safety, tolerability, and feasibility of psilocybin therapy for depression and anxiety in people with PD.	Completed
NCT06455293	Psilocybin (Two doses)	PD and Depression	Phase 2	This clinical study evaluates whether individuals with PD and comorbid depression exhibit symptomatic improvement following psilocybin therapy. Utilizing a multimodal approach—including clinical assessments, advanced neuroimaging, non-invasive brain stimulation, and peripheral blood draws—the researchers will track changes over multiple time points.	Recruiting
PsyPal project	Psilocybin (Multisite)	Chronic obstructive pulmonary disorder (COPD), multiple sclerosis (MS), amyotrophic lateral sclerosis (ALS) and PD	Phase 2	The trial will investigate whether psilocybin therapy can help psychological and existential distress in patients suffering from one of four different progressive diseases.	Initiated

## Data Availability

No new data were created or analyzed in this study.

## Abbreviations

The following abbreviations are used in this manuscript:

5-HT	5-hydroxytryptamine (serotonin)
6-OHDA	6-hydroxydopamine
AAV	Adeno-associated viral vector
AC1/Cg1	Anterior cingulate cortex, subdivision 1
AC2/Cg2	Anterior cingulate cortex, subdivision 2
AMPA	α-Amino-3-hydroxy-5-methyl-4-isoxazolepropionic acid
ATP8A1	ATPase phospholipid transporting 8A1
BDNF	Brain-derived neurotrophic factor
BOLD	Blood-oxygen-level-dependent
CADPS2	Calcium dependent secretion activator 2
Cg	Cingulate cortex
DA	Dopamine/dopaminergic
DLB	Dementia with Lewy bodies
dlPFC	Dorsolateral prefrontal cortex
DMN	Default mode network
E/I	Excitatory/inhibitory
EPSC	Electrically evoked excitatory postsynaptic current
GABA	Gamma-aminobutyric acid
GABARAP	Gamma-aminobutyric acid receptor-associated protein
GAD	Glutamic acid decarboxylase
Glu	Glutamate
GluA1/GluA2	AMPA receptor subunits 1 and 2
GluN1/GluN2A/B	NMDA receptor subunits 1, 2A, and 2B
IL	Infralimbic cortex
L5/6	Layer 5/Layer 6
LC	Locus coeruleus
LBDs	Lewy body dementias
LTP	Long-term potentiation
mEPSC	Miniature excitatory postsynaptic current
mPFC	Medial prefrontal cortex
MPTP	1-Methyl-4-phenyl-1,2,3,6-tetrahydropyridine
mTOR	Mammalian target of rapamycin
NA	Noradrenaline
NAC	Non-amyloid-β component
NMDA	N-methyl-D-aspartate
NSF	N-ethylmaleimide-sensitive factor
PD	Parkinson’s disease
PDD	Parkinson’s disease dementia
PD-MCI	Parkinson’s disease-associated mild cognitive impairment
PET	Positron emission tomography
PFF	Pre-formed fibril
PrL	Prelimbic cortex
PSD-95	Postsynaptic density protein 95
PV	Parvalbumin
rab27B	Ras-related protein
RN	Raphe nuclei
rs-fMRI	Resting-state functional magnetic resonance imaging
SN	Substantia nigra
SNc	Substantia nigra pars compacta
SNARE	Soluble N-ethylmaleimide-sensitive factor attachment protein receptor
SNCA	Synuclein alpha gene
SOC	α-Synuclein origin site and connectome
SPN	Striatal spiny projection neuron
SST	Somatostatin
SV	Synaptic vesicle
SV2A/2B	Synaptic vesicle glycoprotein 2A/2B
TrkB	Tropomyosin receptor kinase B
VAMP2	Vesicle-associated membrane protein 2
VGAT	Vesicular GABA transporter
VGLUT1	Vesicular glutamate transporter-1
VGLUT2	Vesicular glutamate transporter-2
vmPFC	Ventromedial prefrontal cortex
VTA	Ventral tegmental area
WT	Wild-Type
α-Syn	α-Synuclein
1H-MRS	Proton magnetic resonance spectroscopy
